# Differences in the quality of interpersonal care in complementary and conventional medicine

**DOI:** 10.1186/1472-6882-10-63

**Published:** 2010-11-04

**Authors:** André Busato, Beat Künzi

**Affiliations:** 1 Institute for Evaluative Research in Medicine, University of Bern, Stauffacherstrasse 78, CH-3014, Bern, Switzerland; 2 Swisspep - Institute for Quality and Research in Healthcare, Postgasse 17 - CH 3011 Bern, Switzerland

## Abstract

**Background:**

The study was part of a nationwide evaluation of complementary and alternative medicine (CAM) in Swiss primary care. The aim of the study was to compare patient-physician relationships and the respective patient-reported relief of symptoms between CAM and conventional primary care (COM).

**Methods:**

A comparative observational study in Swiss primary care with written survey completed by patients who visited a GP one month earlier. 6133 patients older than 16 years of 170 certified CAM physicians, of 77 non-certified CAM physicians and of 71 conventional physicians were included. Patients completed a questionnaire aimed at symptom relief, patient satisfaction, fulfilment of expectations, and quality of patient-physician interaction (EUROPEP questionnaire).

**Results:**

CAM physicians treated significantly more patients with chronic conditions than COM physicians. CAM Patients had significant higher healing expectations than COM patients. General patient satisfaction was significantly higher in CAM patients, although patient-reported symptom relief was significantly poorer. The quality of patient-physician communication was rated significantly better in CAM patients.

**Conclusions:**

The study shows better patient-reported outcomes of CAM in comparison to COM in Swiss primary care, which is related to higher patient satisfaction due to better patient-physician communication of CAM physicians. More effective communication patterns of these physicians may play an important role in allowing patients to maintain more positive outcome expectations. The findings should promote formative efforts in conventional primary care to improve communication skills in order to reach the same levels of favourable patient outcomes.

## Background

The trend that patients seek more and more help in complementary and alternative medicine (CAM) is a phenomenon in almost all western healthcare systems. CAM therapies that are used instead of conventional medicine are termed "alternative." CAM therapies used alongside conventional medicine are said to be "complementary." With reference to this merging of CAM with conventional biomedicine (COM) an increasing number of clinicians and researchers uses the term "integrative" medicine. However, it is argued that combination medicine (CAM added to COM) is not integrative as such. Integrative medicine represents a higher-order system of care that emphasizes wellness and healing of the entire person (bio-psycho-socio-spiritual dimensions) as primary goals, drawing on both conventional and CAM approaches in the context of a supportive and effective physician-patient relationship[1]. According to the US National Center for Complementary and Alternative Medicine (NCCAM) CAM therapies can be classified into five categories or domains: 1) alternative medical systems (e.g., homeopathy, naturopathy and traditional Chinese medicine), 2) mind-body interventions, 3) biologically based therapies (e.g., foods, vitamins, herbs), 4) manipulative and body-based methods (e.g., chiropractic, massage) and 5) energy therapies (e.g., Therapeutic Touch, Qigong)[2]. Nowadays, e.g. about three quarters of primary care physicians in Switzerland offer CAM themselves or refer their patients to CAM treatments[3] and more than half of all Canadians use some form of CAM every year[4]. Hence, the magnitude of the use of CAM therapies, the way they are being used, and the lack of clarity on standards of evidence make CAM a rising healthcare issue.

New evidence from research is needed to guide a thoughtful incorporation of concepts, values, and practices from CAM and COM towards an integrative medicine as outlined above. The respective research agenda, which may be similar in many Western countries, encompasses high priority areas for health politicians, professionals and consumers alike (cf. recommendations of IN-CAM cited in[4]: (1) healthcare delivery and policy research, including (a) exploring if and how CAM should be regulated, (b) defining what constitutes acceptable evidence of safety and efficacy, (c) investigating the organization and delivery of integrative healthcare; (2) methodological research, including exploring how best to assess whole systems of care and how to choose patient-, practitioner- and policy-relevant outcome measures; and (3) knowledge transfer, including formal education strategies, the provision of information and dialogue with those who use information in decision-making.

When the Swiss Federal Department of Home Affairs decided in 1998 to add four methods of complementary medicine to the benefit catalogue of basic health insurance for a period of five years, many of these issues entered a public discussion, which left more questions than answers. The choice of the four CAM therapies (homeopathy, anthroposophical medicine, neural therapy, and traditional Chinese herbal medicine) reflected more market forces and lobbying than scientific evidence. The policy at that time was, that reimbursements of expenditures for alternative medicine were covered by basic health insurance only for physicians with appropriate CAM training approved by the Swiss Medical Association. Further, it was decided that a nationwide evaluation of the respective CAM therapies had to be performed allowing an evidence-based decision about longer-term inclusion of CAM procedures in compulsory health plans. Western herbal medicine was also included in the project although such an evaluation was not required by federal legislation (the Swiss Medical association does currently not issue board certification for Western herbal medicine).

Based on the results of this evaluation [5], the Swiss Federal Office of Home Affairs decided in 2005 to withdraw CAM procedures from basic health insurance coverage. The main reason for this decision was the lack of persuasive evidence of cost effectiveness, efficacy, and efficiency.

As part of this project, the goal of this study was to use patients' evaluations as a measure of effectiveness of complementary medicine in primary care. Many articles of that time addressed the methodological difficulties inherent in assessing outcomes of complex interventions in CAM[6], and only sparse evidence from research underscored the role of possible modulating factors on outcomes of individualized care, both in CAM and COM. Since then a growing body of evidence underpins that the quality of the physician-patient relation may influence treatment outcomes[7,8].

Therefore complex methods and multiple measures were proposed to research the therapeutic relationship and its impact on treatment outcomes[9]. In this respect patient expectations of treatment benefit and the fulfilment of patients needs seem to play a crucial role in affecting patient-reported outcomes in CAM and COM. But results from respective trials are inconsistent ranging from surprising evidence from COM, showing that optimistic patient expectations may be related indeed with a significant better survival after 2 months when controlled for other physical and mental characteristics[10] to a recent landmark CAM trial, that could not confirm previous studies, which suggested that treatment expectations and patients' preferences might predict outcomes of acupuncture for chronic back pain [11]. These actual findings suggest that the relationship between patient expectations and outcomes may be more complex than previously believed.

Another question is, if and how clear treatment preferences, which are typical in CAM patients, may predict treatment outcomes. Several studies suggest that expectations of positive outcomes are associated with greater treatment satisfaction[12]. Therefore elucidating, understanding, and ultimately negotiating the factors that affect the individual patients' perceptions remains important, because these perceptions affect treatment expectations, which may predict treatment outcomes and hence satisfaction with care. CAM and COM researchers nowadays recommend the use of individualized measures to assess unique patient-centred outcomes for each research participant, and measures to assess the context of healing and the process of healing[9].

The unique situation in Switzerland at the time of the study that offered CAM and COM care within a well defined health care setting allowed the investigation of the influence of further factors such as e.g. additional training of primary care physicians in different CAM therapies, the use of different CAM or COM interventions or the fulfilment of patients' healing expectations on health outcomes of a wide range of symptoms.

The specific research question was: How do patient-physician relationships and respective patient-reported relief of symptoms differ in CAM and COM in primary care? Based on available evidence of today we hypothesize that physicians who are able to developed a strong patient-physician relation in the sense of a working partnership, e.g. by effective communication skills, i) involve more patients in terms of a shared-decision-making about therapeutic options, and ii) set more realistic patient expectations, which are agreed on by patient and physician that might lead to a higher fulfilment of respective patient's expectations, and iii) therefore have patients with better satisfaction with treatment, iv) which ultimately may be associated with a better outcome in terms of patient-reported symptom relief.

## Methods

### Physicians and Patients

The study was designed as a comparative observational study of patients seeking conventional or CAM treatment in Swiss primary care. The target population included all adult primary care patients in Switzerland. The sample population consisted of a convenience sample of 6133 adult patients (> 16 years) treated by 319 physicians all over the country during a 12 month period in 2002 and 2003. Physicians were enrolled on a voluntarily basis and were reimbursed with 500 Swiss Francs (330 €) for their expenditures.

Three groups of physicians were defined for the study based on self-declared medical activity and professional qualification:

- **COM physicians**: Physicians performing no CAM procedures (conventional primary medical care physicians).

- **Noncertified CAM physicians**: Physicians performing CAM and COM procedures without professional certification in CAM and without patient reimbursement of expenditures for CAM procedures by basic health insurance.

- **Certified CAM physicians**: Dual-trained Physicians [13] performing CAM and COM procedures with CAM certificates provisionally recognized by basic health insurance (homeopathy, anthroposophical medicine, neural therapy, traditional Chinese medicine).

Physicians performing Western herbal medicine were part of the noncertified CAM group as the Swiss Medical Association does not issue board certification for this medical discipline.

Only physicians working as primary care providers in ambulatory practice at least two working days per week and with at least five completely documented consultations during the twelve month study period were eligible for our study.

Patient groups were defined according to the same classification. Physicians were instructed to sample consecutive patients consulting their practices on four given days during a 12 month period (one day each in spring, summer, fall and winter). Sampling days were selected at random by the study coordination and were equally distributed across weekdays. Patients were informed about the study by leaflets, provided written informed consent, and were asked to fill out forms about demographic and health status information, treatment related expectations, and frequency and reasons for encounter. Physicians documented the same consultations with reference to symptoms, diagnoses, duration of problems, comorbid conditions, and diagnostic and therapeutic procedures. Physicians had no access to forms completed by patients. The ethics committee of the Canton of Bern raised no objection to the study.

### Outcome

Outcomes were obtained from questionnaires mailed to patients three weeks after the first recorded consultation. Outcomes included self-reported resolution of symptoms, fulfilment of expectations, satisfaction with treatments and a broad range of important interpersonal and organisational dimensions of primary care using the EUROPEP [14] questionnaire. This questionnaire has 23 questions, each with a five point answer scale ranging from poor to excellent covering the following dimensions:

- **Relation and communication **(6 questions)

- **Medical care **(5 questions)

- **Information and support **(4 questions)

- **Continuity and cooperation **(2 questions)

- **Organization of care, including availability and accessibility **(6 questions)

Outcomes measures included therefore data on experience with the treatment process and the related health effects. A time lag of approximately one month appeared therefore to be reasonable to cover both of these domains by trying to minimize recall bias about the recollection of consultation characteristics and by maximizing treatment effects as perceived by patients.

An SF-36 questionnaire [15] was also included in the mailed survey. The goal was to obtain valid estimates of physical and mental well-being in the study population in order to scale potential differences of outcomes. The SF-36 data were therefore not considered as an outcome but as attributes of patient populations. Mental and physical health scores (MCS, PCS) were calculated according to Ware [15]. Questionnaires were provided in German, French, or Italian depending on patient mother tongue. Obtaining a consistent population of patients returning questionnaires with respect to the time between the first recorded consultation and filling out the respective questionnaire was considered critical. Therefore, no reminder letters were sent to nonresponders.

### Comparison

CAM was defined within the project as medical procedures provided by conventionally trained and licensed physicians that usually are not taught in Swiss medical schools. The study was based on concepts of health service research in a primary care setting. Therefore CAM was compared with COM as a whole, and a wide range of indications and procedures were included and evaluated within the project.

### Data analysis

Data analysis included descriptive and analytical procedures. Chi-Square tests were used for univariable analyses and linear- and logistic models for multivariable analyses. Linear models were used to analyse continuous target variables (age, consultation time, SF-36 data). Ordinal outcomes were reduced to binary scales with the most favourable answer category coded as one and all other non-missing categories as zero. These data were analyzed using multivariable logistic regression models and two indicator variables were created in order to allow comparisons between COM and both groups of CAM physicians. Covariables of multivariable models were defined a priori and were aimed to adjust for demographic factors of patients (age and gender). Additional analyses stratified by educational status of patients, by patient perceived disease severity and by disease duration (chronic/non-chronic) were performed in order to ensure the validity of this model. All analytical procedures accounted for clustering of observations at the practice level using Taylor series expansion procedures for 2*2 tables and mixed effects models [16,17] for multivariable procedures; 95% confidence intervals (95% CI) of means, proportions, and odds ratios were calculated accordingly. Model fit procedures for both linear and logistic models provided no indication of a violation of basic assumptions of the respective models and both procedures accounted for reasonable amounts of variation of outcome variables.

Additional demographic data of all patients of the participating physicians were obtained from the data pool of Swiss health insurers (Santésuisse), and health-status and outcome information was obtained from another study performed in the setting of Swiss primary care [18]. This information allowed an assessment of the generalisabilty of our data. The level of significance was set at p < 0.05 throughout the study and SAS 9.2 (SAS Institute Inc., Cary, NC, USA) was used for all calculations.

## Results

11,615 consultations of patients older than 16 years were documented in 318 primary care practices (71, 77 and 170 for COM, noncertified CAM and certified CAM respectively). The sample of physicians included 5.3% of all primary providers and of 29% of all certified CAM physicians in Switzerland in 2002. 102 physicians were certified in homeopathy, 48 in traditional Chinese medicine, 22 in anthroposophical medicine, 17 in neural therapy, and 19 physicians had multiple CAM certificates.

Seventy-six patients (0.7%) filled out forms during the consultation but declined further participation in the study. Therefore, 11,539 questionnaires were mailed to patients three weeks later. The proportion of questionnaires returned by patients within one month was 53.2% (n = 6133).

Questionnaires were completed by patients on average 26 days after the initial consultation. Compliance was significantly higher for patients living in German-speaking parts of Switzerland, for female patients, for patients older than 30 years, patients with problems lasting longer than three months, and for patients treated by certified CAM physicians. The comparison of responders' demographics with the respective information derived from the health insurers' data pool indicated that female and younger patients were overrepresented, i.e. 68.5% females in the sample vs. 65.0% in the data pool, and 48.9 years of age vs. 54.2 years respectively. Additional information available from another large Swiss study performed in conventional primary care [18,19] indicated almost identical patterns for general health in COM patients.

All following data refer to patients with complete consultation data and who returned the postal questionnaire.

### Procedures and management of disease

Gender distribution and gender-adjusted ages of patients were significantly different between responder groups (p < 0.01 for gender and age), and patients of certified CAM physicians were significantly better educated (p < 0.01) (Table 1). No significant differences between groups were observed for how patients rated their general health prior to the consultation (p = 0.12). But significant differences were observed for treatment expectations. With reference to COM, patients of both groups of CAM physicians more frequently expected healing of their symptoms (p = 0.04 for noncertified CAM, p < 0.01 for certified CAM). Expectations of symptom relief also were significantly different between certified CAM and COM (p < 0.01), but no significant difference was seen between noncertified CAM and COM (p = 0.10).

**Table 1:** Patient attributes and consultation times

Type of treatment	COM	Noncertified CAM	Certified CAM
Age of patients in years*	53.9	52.6	49.4
Proportion of female patients*	59.2%	64.4%	74.0%
Proportion of patients with higher education^a*^	24.7%	23.5%	30.0%
Treatment expectations*			
- Healing	49.6%	49.6%	58.3%
- Symptom relief	41.5%	40.8%	44.6%
Proportion of chronic patients^b*^	46.2%	46.9%	62.1%
Proportion of patients with severe symptoms*	9.8%	12.7%	17.9%
Consultation time (LSM)^c*^	17.3 min	20.7 min.	28.3 min.
- non chronic patients*	16.9 min.	19.3 min.	25.7 min.
- chronic patients*	17.7 min.	22.0 min.	29.5 min.

a College or university degrees

b Disease duration more than 3 months

c Gender- and age-adjusted least square means

* Significant differences between groups (p < 0.05, chi-square tests for categorized outcomes, linear models for continuous outcomes)

With reference to COM, age and gender adjusted proportions of chronically ill patients, defined as having health problems lasting longer than 3 months, was significantly higher for certified CAM physicians (p < 0.01) and significantly lower for noncertified CAM (p = 0.01). Certified CAM physicians documented significantly higher loads of patients with severe symptoms (p < 0.01) but no significant difference was seen for noncertified CAM (p = 0.59). Average consultation time was 22.6 minutes and differed significantly between groups (Table 1). There were considerable effects of disease duration on consultation times. Consultations for chronic patients lasted longer in all study groups. However, first order interaction terms indicated that consultation length of chronic patients of certified CAM physicians was significantly and disproportionately longer than in other groups.

Patients were treated for various medical conditions [20] by applying a broad range of treatment modalities (Table 2). The frequencies of main diagnoses according the ICD10 main chapters are given in Table 3. Certified CAM physicians treated considerably more patients with musculoskeletal, mental and behavioural problems, and diseases of the nervous system, whereas COM physicians treated more patients with cardiovascular problems. No significant differences between groups were observed for average number of comorbid conditions of patients. Charlson comorbidity indices [21] as a measure of mortality risks were calculated based on ICD10 codes. It appeared that COM physicians treated significantly more patients with Charlson comorbidity indices greater than zero (6.5%, 9.4%, and 11.4% for certified CAM, noncertified CAM, and COM respectively). Certified CAM physicians were using solely COM procedures in 17% and noncertified CAM physicians in 60% of their consultations.

**Table 2:** Distribution of therapeutic procedures across patient groups

	COM		Noncertified CAM		Certified CAM	
Type of treatment	n	%	N	%	n	%
COM	1199	88.0	826	60.0	579	17.1
Homeopathy	2	0.1	39	2.8	838	24.7
CAM & COM	9	0.7	157	11.4	435	12.8
No treatment	128	9.4	121	8.8	226	6.7
Multiple CAM			28	2.0	351	10.3
Acupuncture	6	0.4	24	1.7	344	10.1
Anthroposophical medicine			94	6.8	215	6.3
Neural therapy	-	-	9	0.7	182	5.4
Other	17	1.2	29	2.1	127	3.7
Western Herbal medicine	2	0.1	49	3.6	26	0.8
TCM	-	-			71	2.1

Total	1363	100.00	1376	100.00	3394	100.00

**Table 3:** Distribution of main diagnoses (frequency in percent per ICD10-Chapters across patient groups)

ICD10 Chapters	COM	Noncertified CAM	Certified CAM
M00-M99 Diseases of the musculoskeletal system and connective tissue	17.5	20.8	21.0
I00-I99 Diseases of the circulatory system	17.7	11.0	6.7
J00-J99 Diseases of the respiratory system	9.9	9.3	10.2
F00-F99 Mental and behavioral disorders	8.2	7.4	11.6
K00-K93 Diseases of the digestive system	6.3	4.9	5.9
S00-T98 Injury, poisoning and certain other consequences of external causes	7.6	7.0	4.5
R00-R99 Symptoms, signs and abnormal clinical and laboratory findings, not elsewhere classified	3.5	4.6	6.7
N00-N99 Diseases of the genitourinary system	3.1	5.5	5.2
G00-G99 Diseases of the nervous system	2.6	2.5	6.3
L00-L99 Diseases of the skin and subcutaneous tissue	3.4	3.9	4.9
E00-E90 Endocrine, nutritional and metabolic diseases	5.8	4.9	3.1
Z00-Z99 Factors influencing health status and contact with health services	5.2	4.9	2.2
C00-D48 Neoplasms	2.1	3.1	2.7
No diagnosis specified	2.2	3.5	2.4
A00-B99 Certain infectious and parasitic diseases	1.7	3.0	2.6
H60-H95 Diseases of the ear and mastoid process	1.8	1.7	1.9
D50-D89 Diseases of the blood and blood-forming organs and certain disorders involving the immune mechanism	0.4	1.1	0.8
H00-H59 Diseases of the eye and adnexa	0.6	0.3	0.7
O00-O99 Pregnancy, childbirth and the puerperium	0.1	0.4	0.3
Q00-Q99 Congenital malformations, deformations and chromosomal abnormalities	0.1	0.2	0.2

### Patient evaluations (Table 4)

Significant differences between patient groups were observed for unadjusted proportions of patient-reported resolution of symptoms since the previously recorded consultation. With reference to COM, age- and gender-adjusted odds of complete symptom resolution were significantly lower in certified CAM (OR 0.71) and non-significantly different for noncertified CAM (OR 1.1). Resolution of symptoms was significantly associated with age. Unadjusted proportions of fulfilment of treatment-related expectations appeared to be significantly different between groups. However, age- and gender-adjusted odds ratios for complete fulfilment of expectations yielded no significant differences between CAM and COM patients (OR certified CAM: 1.00, noncertified CAM: 1.01), although age appeared as a significant factor. Unadjusted proportions of general treatment satisfaction were also different between groups; 51% of patients in both CAM groups appeared to be very satisfied with their treatments, in contrast to 43% in COM patients. But multivariate logistic modelling with reference to COM revealed different results. Odds of complete satisfaction were significantly higher only in certified CAM (OR 1.12), and not for noncertified CAM patients (OR 1.01). Additionally, a significant association was seen for gender of patients.

**Table 4:** Patient outcomes (symptom resolution, fulfilment of expectations, treatment satisfaction)

	COM			Noncertified CAM			Certified CAM		
	N	%	95% CI	N	%	95% CI	n	%	95% CI
Symptom resolution									
Complete resolution	358	27.6	24.4-30.7	338	25.7	22.9-28.5	616	18.6	16.8-20.5
Much better	392	30.2	27.5-32.9	410	31.2	28.3-34.1	1358	41.1	39.0-43.1
Better	219	16.9	14.9-18.9	265	20.2	17.7-22.7	767	23.2	21.7-24.7
Equal	300	23.1	20.9-25.3	284	21.6	18.8-24.4	513	15.5	13.8-17.2
Worse	22	1.7	0.9-2.5	16	1.2	0.7-1.8	48	1.5	1.1-1.8
Unbearable	7	0.5	0.2-0.9	1	0.1	0.0-0.2	4	0.1	0.0-0.2

									
**Fulfilment of expectations**									

Complete	409	32.6	29.2-35.9	439	34.3	30.9-37.6	1112	33.9	32.0-35.9
mostly yes	578	46.0	42.9-49.2	598	46.7	43.8-49.6	1713	52.3	50.1-54.5
mostly not	196	15.6	13.5-17.7	189	14.8	12.4-17.1	362	11.1	9.6-12.5
Not at all	73	5.8	4.5-7.1	55	4.3	3.1-5.5	89	2.7	2.0-3.4

									
**Treatment satisfaction**									

very satisfied	549	43.4	40.5-46.3	651	50.6	46.7-54.6	1693	51.2	49.2-53.2
mostly satisfied	571	45.1	42.3-48.0	500	38.9	35.7-42.1	1377	41.6	39.9-43.3
mostly not satisfied	119	9.4	7.9-10.9	110	8.6	7.1-10.0	212	6.4	5.2-7.6
Not satisfied at all	26	2.1	1.2-2.9	25	1.9	1.1-2.8	25	0.8	0.4-1.1

**Figure 1:**
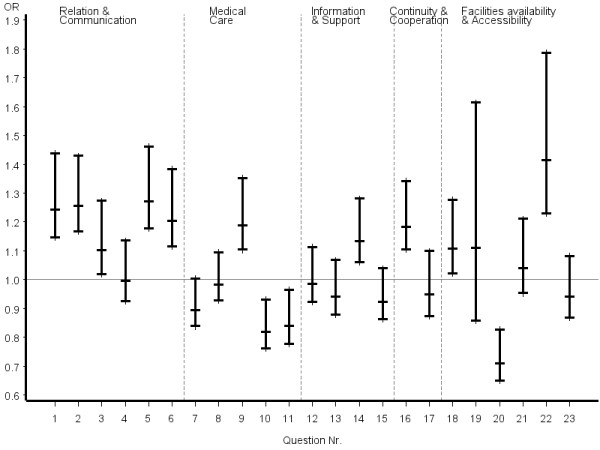
**..Europep questionnaire, odds ratios of most favourable answer option (certified CAM vs. COM) all patients**. Errorbars denote 95% confidence intervals of odds ratios

The first six questions of the EUROPEP questionnaire (Table 5, Figure 1), covering relation and communication, revealed consistent answer patterns with reference to COM patients. The most favourable answers were significantly more frequently given in both groups of CAM patients, with a trend toward better outcomes in certified CAM. Less consistent answers were seen for questions related to medical care. Thoroughness was rated significantly higher by both CAM groups, whereas symptom relief, physical examination during the consultation, and offering services for preventing diseases were significantly better rated by COM patients. Only one question regarding information and support, referring to help with emotional problems, was rated significantly better by certified CAM patients. Within the dimension of continuity and cooperation, only the item "knowing what the physician did or said during earlier contacts" was significantly perceived as superior by certified CAM patients.

**Table 5:** Patient evaluation of COM and CAM, EUROPEP questionnaire

Nr	Questions/items	% of best answer				
		COM	Noncertified CAM		Certified CAM	
	**Relation and communication**		**%**	**OR**	**%**	**OR**

1.	Making you feel you had time during consultation?	61.7	70.5	1.1	72.1	1.3*
2.	Interest in your personal situation?	60.3	68.9	1.1	71.3	1.3*
3.	Making it easy for you to tell him or her about your problem?	62.9	70.4	1.1	68.7	1.1*
4.	Involving you in decisions about your medical care?	58.4	66.2	1.2*	61.7	1.0
5.	Listening to you?	67.1	75.0	1.1	77.0	1.3*
6.	Keeping your records and data confidential?	75.4	78.1	1.0	81.8	1.2*

	**Medical care**					

7.	Quick relief of your symptoms?	27.6	28.7	1.1	24.9	0.9
8.	Helping you to feel well so that you can perform your normal daily activities?	41.2	44.4	1.1	42.9	1.0
9.	Thoroughness?	56.5	67.3	1.1*	68.6	1.2*
10.	Physical examination of you?	52.6	58.5	1.2*	48.2	0.8*
11.	Offering you services for preventing diseases (screening, health checks, immunizations, ...)	48.7	49.3	1.1	43.0	0.9*

	**Information and support**					

12.	Explaining the purpose of tests and treatments?	60.2	65.5	1.1	63.0	1.0
13.	Telling you what you wanted to know about your symptoms and/or illness?	60.2	69.7	1.3*	63.3	1.0
14.	Helping you deal with emotional problems related to your health status?	49.7	57.6	1.1	58.7	1.2*
15.	Helping you understand the importance of following his or her advice?	51.0	52.7	1.1	49.0	0.9

	**Continuity and cooperation**					

16.	Knowing what s/he had done or told you during earlier contacts?	53.4	58.9	1.0	61.9	1.2*
17.	Preparing you for what to expect from specialist or hospital care?	55.7	59.8	1.1	56.7	1.0

	**Facilities availability and accessibility**					

18.	The helpfulness of the staff (other than the doctor)?	66.1	68.3	1.0	70.6	1.1*
19.	Getting an appointment to suit you?	1.2	1.6	1.0	1.8	1.2
20.	Getting through to the practice on telephone?	72.1	70.9	1.1	61.9	0.7*
21.	Being able to speak to the general practitioner on the telephone?	58.3	60.8	1.0	61.5	1.1
22.	Waiting time in the waiting room?	38.1	41.7	0.9	53.6	1.5*
23.	Providing quick services for urgent health problems?	71.6	73.0	1.0	71.4	1.0

* Significant odds ratio (p < 0.05) with reference to COM group in a multivariable logistic model with age and gender as cofactors (PROC NLMIXED, SAS 9.2)

**Figure 2:**
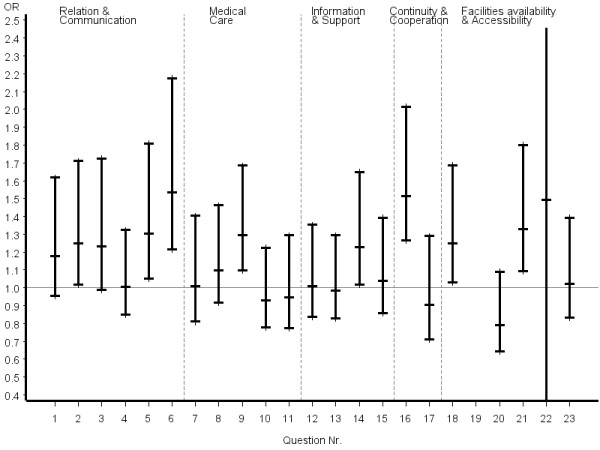
**..Europep questionnaire, odds ratios of most favourable answer option (certified CAM vs. COM) Stratified analysis of patients with severe conditions (patient rated)**. Errorbars denote 95% confidence intervals of odds ratios

**Figure 3:**
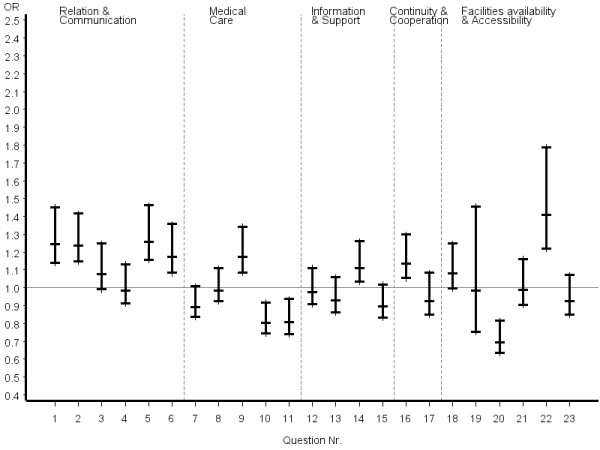
**..Europep questionnaire, odds ratios of most favourable answer option (certified CAM vs. COM) Stratified analysis of patients with non-severe conditions (patient rated)**. Errorbars denote 95% confidence intervals of odds ratios

**Figure 4:**
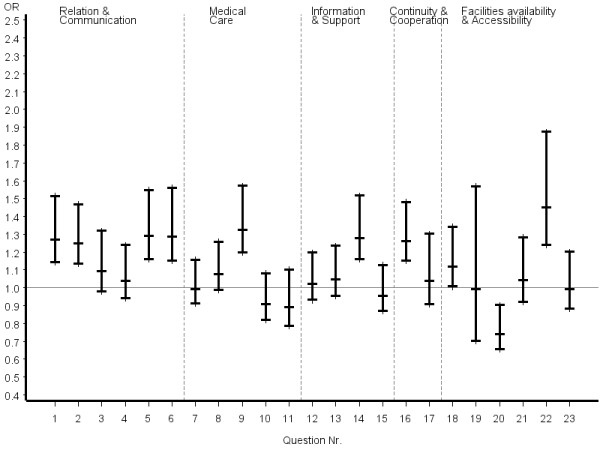
**..Europep questionnaire, odds ratios of most favourable answer option (certified CAM vs. COM) Stratified analysis of patients with chronic conditions**. Errorbars denote 95% confidence intervals of odds ratios

**Figure 5:**
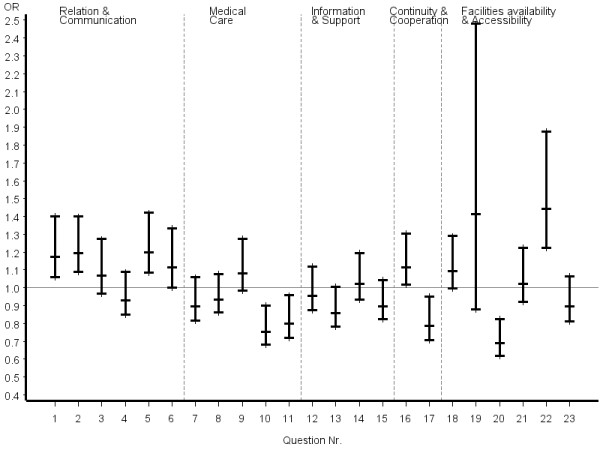
**..Europep questionnaire, odds ratios of most favourable answer option (certified CAM vs. COM) Stratified analysis of patients with non-chronic conditions**. Errorbars denote 95% confidence intervals of odds ratios

**Figure 6:**
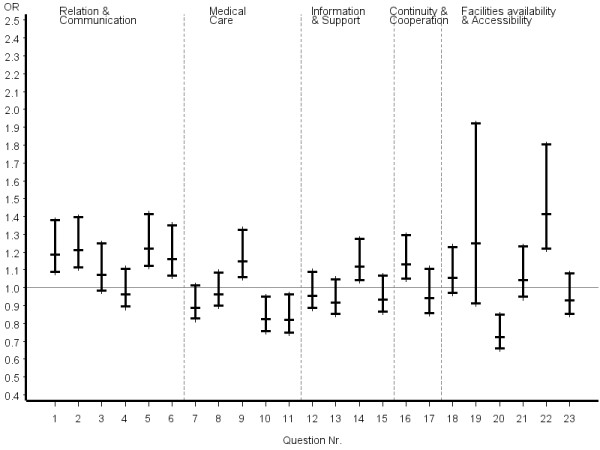
**..Europep questionnaire, odds ratios of most favourable answer option (certified CAM vs. COM) Stratified analysis of patients with university degrees**. Errorbars denote 95% confidence intervals of odds ratios

**Figure 7:**
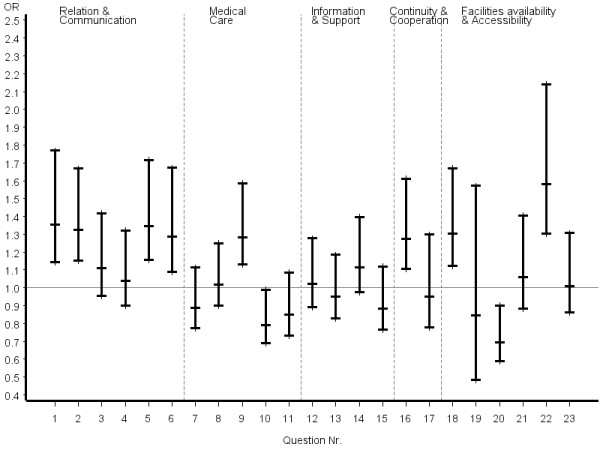
**..Europep questionnaire, odds ratios of most favourable answer option (certified CAM vs. COM) Stratified analysis of patients without higher education**. Errorbars denote 95% confidence intervals of odds ratios

Inconsistent answer patterns across patient groups were also observed for questions referring to organization of care, including availability and accessibility. Helpfulness of staff and waiting time in the waiting room were rated significantly better by certified CAM patients, whereas reaching the practice by telephone was rated significantly poorer. However, it appeared that obtaining suitable appointments was a common problem for all patients in the study. Odds ratios and the related 95% confidence intervals of the most favourable answer option of certified CAM vs. COM physicians stratified by disease severity, chronicity and educational status of patients are given in figures 2, 3, 4, 5, 6, 7. The stratified analyses indicate consistently better outcomes for the domain relation and communication and comparable results for all other domains of the EUROPEP data irrespective of stratification criteria. Cofactor analysis of the EUROPEP data revealed a wide range of significant and potentially relevant associations beyond the immediate scope of this paper. A further interpretation of these findings was therefore omitted.

The SF-36 data indicated significant differences for physical health scores in the three patient populations. Gender and age adjusted PCS values were certified CAM, 49.2; noncertified CAM, 48.0; COM, 48.5. No significant differences were observed for mental health scores: certified CAM, 48.1; noncertified CAM, 48.9; COM, 47.7. The remaining SF-36 data are given in Table 6.

**Table 6:** SF-36 data

Items (0-100)	COM	Non-certified CAM	Certified CAM
Physical functioning*	79.9	80.3	84.0
Role physical	84.3	83.0	82.4
Pain index	65.9	65.0	66.0
General health	61.6	62.3	63.3
Vitality	55.2	55.3	54.9
Social Functioning	78.2	78.6	78.8
Role emotional	89.1	90.1	88.9
Mental health index	69.8	70.8	69.8

^* ^Significantly different (p < 0.05) between groups in a multivariable linear model with age and gender as cofactors

## Discussion

Quality of care was defined based on concepts of structure, process, and outcome [22,23], and the interpretation of the outcome data discriminated between intervention- and process-related items of care [24,25]. Intervention-related items refer to the patient-reported treatment effectiveness in terms of symptom relief and matched expectations, and process-related items refer to quality of interpersonal care.

Based on these concepts, distinct patterns were observed in how quality of care was reported by COM and CAM patients. The observation that CAM patients experienced poorer relief of symptoms is likely associated with higher proportions of patients with chronic and apparently more severe disease conditions. However, self-rated general health prior to the consultation and at follow-up point to paradoxical findings. Despite a higher load of chronic and more severe health problems, CAM patients had equal or even better general health and higher SF-36 scores than COM patients. It can be speculated that these findings are associated with different mechanisms for coping with illness and disease, or that they are the result of higher mortality risks observed in COM patients.

Outcomes are not only associated with the effectiveness of a treatment but with a variety of psychological and societal factors [26] embedded in a dynamic process of interaction between patient and physician that involves expectations, beliefs, and behavioural factors of patients and physicians [27].

In this framework, our study supports the hypothesis that patients treated by certified CAM physicians have higher levels of treatment satisfaction, and the results indicate that this favourable outcome is associated with higher treatment expectations. Epistemological doctrines established in the 17th century[28], applied and refined much later in psychological and educational research, may provide explanatory models for these findings. As mentioned before positive expectations may enhance the probability of a more favourable outcome [29]; the concepts of "self-fulfilling prophecy" and "interpersonal expectancy effects" [30] resulted from this work. These concepts may have direct application to our data in the sense that interpersonal expectancy effects may also emerge when physicians confirm and support expectations of their patients. It therefore can be hypothesized from our empirical findings that positive outcomes in CAM are linked to the expectation that CAM treatments provide a better fulfilment of patients needs. The results of the EUROPEP questionnaire support these hypotheses insofar as they indicate that the apparent patient-reported effectiveness of CAM treatments is also linked to more effective communication and better information and continuity of care, but not to patient experiences directly related to medical interventions. Therefore, it appears that interpersonal elements of the quality of care have high priority for CAM patients and are valued as more important than elements associated with specific treatment effects. However, the fact that expectations of CAM patients are less vague than those of COM patients [31]--they are more clearly focused on a specific CAM treatment approach[32]--makes it easier to get fulfilment. It is however important to note that expectations are related to experience and use of CAM [33] and expectancy effects may also differ across specific CAM disciplines.

These results confirm other observations that patients clearly distinguish between task and affective elements of care [25]. Information giving appears to have a key role in this context [34]. Better patient outcomes are generally associated with longer consultation times [35], although there is some debate about which aspects of the behaviour of physicians are major determinants of patient-based evaluations in primary care. The observation of longer consultation times of certified CAM physicians with patients with more chronic conditions or psychiatric diseases, i.e. with less objectively measurable indicators of symptoms, indicates that CAM physicians recognize that such patients are more sensitive to communication [36] and that they should be approached differently.

In summary, differences in the patient-reported effectiveness of CAM and COM seem to be based on more effective ways of communication of certified CAM physicians, which might include also more positive treatment-related outcome expectations. Thus, from a patient perspective CAM physicians provide a more effective type of care, even though the specific clinical effectiveness of CAM procedures is questionable [37,38]. From a health system perspective, the extent to which such differences in patient-physician interactions are more likely to produce other patterns of diagnoses and a different use of health care resources in CAM than in conventional primary care remains an open question.

### Limitations and strengths

The study was planned as a purely observational assessment of patient outcomes and consequently suffers from a number of limitations common to this type of research. These include selection bias of physicians and both response and recall bias of patients who decided to complete the questionnaires. It can be assumed that the motivation among participating physicians was different, since CAM physicians were under more pressure to demonstrate effective methods--which was not the case for COM physicians. It can only be speculated that the motivation of COM physicians is more attributable to a general interest in primary care research. In a strict sense, the generalisability of our results is therefore reduced to physicians with these distinct motivations. Satisfied and dissatisfied patients have different compliance in completing questionnaires. It is therefore very likely that the results are positively biased because satisfied patients are more likely to return the questionnaires [39,40]. However, patients' evaluations of care offer not only unique subjective information, which is otherwise not available, but also provide valid estimates of their experiences and respective satisfaction in a primary care setting [41,42]. A further limitation refers to the short follow-up period of the study, which prevents quality assessments of ongoing relationship-building processes between patient and physician, and the definition of therapeutic relationships. We did not account for other than demographic factors in the statistical analysis. The decision to use such a parsimonious model is based on earlier research[32] indicating particular population characteristics and motivations inherent to patients using CAM and preliminary analyses indicated considerable collinearity between these variables and patient group. Stratification was therefore used to document effects of potential confounders. These difficulties to adequately control for confounding in our study point to the limitations of quantitative methods when investigating the role of psycho-social effects in clinical research that is aimed to study the effectiveness of CAM. It may also be argued that the analysis of the EUROPEP data needs adjustment for the problem of multiple tests. The literature in this field is inconclusive[43] and the decision whether to view the EUROPEP data as a group or as individual questions remains arbitrary. We tend to consider each question as a single hypothesis and promote therefore a more individualistic view.

It may also be criticized that outcomes were dichotomized into the best possible and all other answer options. This approach is based on a commonly applied concept that standards of excellence attained by top performers should be used as benchmarks of quality in the health care sector[44]. Furthermore considerable ceiling effects were detected when using different pooling procedures particularly for the EUROPEP data.

Strengths of the study are related to the fact that all physicians shared the same medical background--i.e. all physicians were trained and certified in conventional medicine, and CAM physicians were dual-trained within a well-defined national framework. Furthermore, an internationally validated patient survey instrument [45] was used to assess the quality of the physician-patient relationship. Other strengths include the availability of billing data that allowed further validations of patient demographics, which indicated a reasonable fit between the overall population and the sampled data.

## Conclusion

Given the rising age and increasing morbidity and consumerism of the population, efficient management of high--if not unrealistic--patient expectations becomes a core function of primary care. Previous empirical research has underscored the importance the quality of the physician-patient relationship by demonstrating its association with important outcomes, including adherence to medical advice [46] and satisfaction with care. However, few studies have had the benefit of longitudinal data to verify the sequencing of effects between relationship quality and outcomes [47]. Our study therefore provides empirical evidence that the patient-reported effectiveness of CAM in Swiss primary care is related to higher patient satisfaction due to better patient-physician communication of certified CAM physicians. More effective communication patterns of these physicians also may play an important role in allowing patients to maintain more positive outcome expectations. The findings should promote formative efforts in conventional primary care to improve communication skills in order to reach the same levels of favourable patient outcomes.

## Competing interests

The authors declare that they have no competing interests.

## Authors' contributions

AB obtained the mandate to conduct the study, he performed all statistical procedures and wrote a first draft of the manuscript. BK provided considerable input as a primary care physician and completed the manuscript in this respect. Both authors read and approved the final manuscript.

## Pre-publication history

The pre-publication history for this paper can be accessed here:

http://www.biomedcentral.com/1472-6882/10/63/prepub

## References

[B1] Barrett B Marchand L Scheder J Plane MB Maberry R Appelbaum D Rakel D Rabago D Themes of holism, empowerment, access, and legitimacy define complementary, alternative, and integrative medicine in relation to conventional biomedicine J Altern Complement Med 2003 9 6 937 947 10.1089/107555303771952271 14736364

[B2] What is complementary and alternativew medicine (CAM)? http://nccam.nih.gov/health/whatiscam/

[B3] Deglon-Fischer A Barth J Ausfeld-Hafter B [Complementary and alternative medicine in primary care in Switzerland] Forsch Komplementmed 2009 16 4 251 255 10.1159/000207970 19729936

[B4] Boon HS Verhoef MJ Vanderheyden LC Westlake KP Complementary and alternative medicine: a rising healthcare issue Healthc Policy 2006 1 3 19 30 19305666 PMC2585340

[B5] Melchart D Mitscherlich. F Amiet M Eichenberger R Koch P Programm Evaluation Komplementärmedizin (PEK) Schlussbericht 2005 Swiss Federal Office of Public Health

[B6] Mason S Tovey P Long AF Evaluating complementary medicine: methodological challenges of randomised controlled trials BMJ 2002 325 7368 832 834 10.1136/bmj.325.7368.832 12376448 PMC1124333

[B7] Long AF Mercer G Hughes K Developing a tool to measure holistic practice: a missing dimension in outcomes measurement within complementary therapies Complement Ther Med 2000 8 1 26 31 10812757

[B8] MacPherson H Mercer SW Scullion T Thomas KJ Empathy, enablement, and outcome: an exploratory study on acupuncture patients' perceptions J Altern Complement Med 2003 9 6 869 876 10.1089/107555303771952226 14736359

[B9] Verhoef MJ Boon HS Mutasingwa DR The scope of naturopathic medicine in Canada: an emerging profession Soc Sci Med 2006 63 2 409 417 10.1016/j.socscimed.2006.01.008 16487639

[B10] Lee SJ Loberiza FR Rizzo JD Soiffer RJ Antin JH Weeks JC Optimistic expectations and survival after hematopoietic stem cell transplantation Biol Blood Marrow Transplant 2003 9 6 389 396 10.1016/S1083-8791(03)00103-4 12813447

[B11] Sherman KJ Cherkin DC Ichikawa L Avins AL Delaney K Barlow WE Khalsa PS Deyo RA Treatment expectations and preferences as predictors of outcome of acupuncture for chronic back pain Spine (Phila Pa 1976) 2010 35 15 1471 1477 20535051 10.1097/BRS.0b013e3181c2a8d3 PMC2895682

[B12] Marschall-Kehrel D Roberts RG Brubaker L Patient-reported outcomes in overactive bladder: the influence of perception of condition and expectation for treatment benefit Urology 2006 68 2 Suppl 29 37 10.1016/j.urology.2006.02.046 16908338

[B13] Kaptchuk TJ Eisenberg DM Varieties of healing. 2: a taxonomy of unconventional healing practices Ann Intern Med 2001 135 3 196 204 11487487 10.7326/0003-4819-135-3-200108070-00012

[B14] Grol R Wensing M Task force on patient evaluations of general practice care 1999 ISBN 90-76316-11-2 10.1093/fampra/16.1.4 10321388

[B15] Ware JE M K SF-36 Physical & Mental Health Summary Scales: A Manual for Users of Version 1 2001 Second QualityMetric Incorporated Lincoln, Rhode Island

[B16] Singer J Using SAS PROC MIXED to fit multilevel models, hierarchical models, and individual growth models Journal of Educational and Behavioral Statistics 1998 24 4 323 355

[B17] Sheu CF Fitting mixed-effects models for repeated ordinal outcomes with the NLMIXED procedure Behav Res Methods Instrum Comput 2002 34 2 151 157 12109005 10.3758/bf03195436

[B18] Künzi B Zürich Swisspep Qualidoc^® ^gibt Rechenschaft über hausärztliche Wirksamkeit 2004 8

[B19] Künzi B Swisspep Qualidoc^®^: A balanced score card to capture and extend the added values of general practice/family medicine 2004 Houten, NL: Bohn Stafleu van Lohum

[B20] Busato A Donges A Herren S Widmer M Marian F Health status and health care utilisation of patients in complementary and conventional primary care in Switzerland--an observational study Fam Pract 2006 23 1 116 124 Epub 2005 Aug 2022 10.1093/fampra/cmi078 16115833

[B21] Sundararajan V Henderson T Perry C Muggivan A Quan H Ghali WA New ICD-10 version of the Charlson comorbidity index predicted in-hospital mortality J Clin Epidemiol 2004 57 12 1288 1294 10.1016/j.jclinepi.2004.03.012 15617955

[B22] Campbell SM Roland MO Buetow SA Defining quality of care Soc Sci Med 2000 51 11 1611 1625 10.1016/S0277-9536(00)00057-5 11072882

[B23] Donabedian A The quality of care. How can it be assessed? JAMA 1988 260 12 1743 1748 10.1001/jama.260.12.1743 3045356

[B24] Hudak PL Wright JG The characteristics of patient satisfaction measures Spine 2000 25 24 3167 3177 10.1097/00007632-200012150-00012 11124733

[B25] Jung HP Van Horne F Wensing M Hearnshaw H Grol R Which aspects of general practitioners' behaviour determine patients' evaluations of care? Soc Sci Med 1998 47 8 1077 1087 10.1016/S0277-9536(98)00138-5 9723853

[B26] McGeary DD Mayer TG Gatchel RJ Anagnostis C Proctor TJ Gender-related differences in treatment outcomes for patients with musculoskeletal disorders Spine J 2003 3 3 197 203 10.1016/S1529-9430(02)00599-5 14589200

[B27] Crow R Gage H Hampson S Hart J Kimber A Storey L Thomas H The measurement of satisfaction with healthcare: implications for practice from a systematic review of the literature Health Technol Assess 2002 6 32 1 244 12925269 10.3310/hta6320

[B28] Hobbes T (ed) The English Works (Dialogue, Behemont, Rhetoric) 1860 London: John Bohn

[B29] Rosenthal R Rubin DB Interpersonal expectancy effects: The first 345 studies The Behavioral and Brain Sciences 1978 1 377 415 10.1017/S0140525X00075506

[B30] Rosenthal R Experimenter and clinician effects in scientific inquiry and clinical practice Prevention & Treatment 2002 5 1 1 12

[B31] Peck BM Ubel PA Roter DL Goold SD Asch DA Jeffreys AS Grambow SC Tulsky JA Do unmet expectations for specific tests, referrals, and new medications reduce patients' satisfaction? J Gen Intern Med 2004 19 11 1080 1087 10.1111/j.1525-1497.2004.30436.x 15566436 PMC1494793

[B32] Wapf V Busato A Patients motives for choosing a physician: comparison between conventional and complementary medicine in Swiss primary care BMC Complement Altern Med 2007 7 1 41 10.1186/1472-6882-7-4118021390

[B33] Sirois FM Gick ML An investigation of the health beliefs and motivations of complementary medicine clients Soc Sci Med 2002 55 6 1025 1037 10.1016/S0277-9536(01)00229-5 12220087

[B34] Beck RS Daughtridge R Sloane PD Physician-patient communication in the primary care office: a systematic review J Am Board Fam Pract 2002 15 1 25 38 11841136

[B35] Freeman GK Horder JP Howie JG Hungin AP Hill AP Shah NC Wilson A Evolving general practice consultation in Britain: issues of length and context Bmj 2002 324 7342 880 882 10.1136/bmj.324.7342.880 11950738 PMC101402

[B36] Thorne SE Harris SR Mahoney K Con A McGuinness L The context of health care communication in chronic illness Patient Educ Couns 2004 54 3 299 306 10.1016/j.pec.2003.11.009 15324981

[B37] Shang A Huwiler-Muntener K Nartey L Juni P Dorig S Sterne JA Pewsner D Egger M Are the clinical effects of homoeopathy placebo effects? Comparative study of placebo-controlled trials of homoeopathy and allopathy Lancet 2005 366 9487 726 732 10.1016/S0140-6736(05)67177-2 16125589

[B38] Melchart D Streng A Hoppe A Brinkhaus B Witt C Wagenpfeil S Pfaffenrath V Hammes M Hummelsberger J Irnich D Acupuncture in patients with tension-type headache: randomised controlled trial Bmj 2005 331 7513 376 382 10.1136/bmj.38512.405440.8F 16055451 PMC1184247

[B39] A guide to direct measures of patient satisfaction in clinical practice. Health Services Research Group Cmaj 1992 146 10 1727 1731 1596808 PMC1488691

[B40] Mazor KM Clauser BE Field T Yood RA Gurwitz JH A demonstration of the impact of response bias on the results of patient satisfaction surveys Health Serv Res 2002 37 5 1403 1417 10.1111/1475-6773.11194 12479503 PMC1464019

[B41] Turnbull JE Hembree WE Consumer information, patient satisfaction surveys, and public reports Am J Med Qual 1996 11 1 S42 45 8763233

[B42] Wensing M Vedsted P Kersnik J Peersman W Klingenberg A Hearnshaw H Hjortdahl P Paulus D Kunzi B Mendive J Patient satisfaction with availability of general practice: an international comparison Int J Qual Health Care 2002 14 2 111 118 11954680 10.1093/oxfordjournals.intqhc.a002597

[B43] Thompson JR Invited commentary: Re: "Multiple comparisons and related issues in the interpretation of epidemiologic data" Am J Epidemiol 1998 147 9 801 806 9583708 10.1093/oxfordjournals.aje.a009530

[B44] Weissman NW Allison JJ Kiefe CI Farmer RM Weaver MT Williams OD Child IG Pemberton JH Brown KC Baker CS Achievable benchmarks of care: the ABCs of benchmarking J Eval Clin Pract 1999 5 3 269 281 10.1046/j.1365-2753.1999.00203.x 10461579

[B45] Wensing M Mainz J Grol R A standardised instrument for patients' evaluations of general practice care in Europe Eur J Gen Pract 2000 6 82 87 10.3109/13814780009069953

[B46] Safran DG Taira DA Rogers WH Kosinski M Ware JE Tarlov AR Linking primary care performance to outcomes of care J Fam Pract 1998 47 3 213 220 9752374

[B47] Safran DG Montgomery JE Chang H Murphy J Rogers WH Switching doctors: predictors of voluntary disenrollment from a primary physician's practice J Fam Pract 2001 50 2 130 136 11219560

